# An active break program (ACTIVA-MENTE) at elementary schools in Chile: study protocol for a pilot cluster randomized controlled trial

**DOI:** 10.3389/fpubh.2023.1243592

**Published:** 2024-01-08

**Authors:** Tomás Reyes-Amigo, Jessica Ibarra-Mora, Nicolás Aguilar-Farías, Nicolás Gómez-Álvarez, Hernaldo Carrasco-Beltrán, Rafael Zapata-Lamana, Juan Hurtado-Almonácid, Jacqueline Páez-Herrera, Rodrigo Yañez-Sepulveda, Guillermo Cortés, Grissel Rolle-Cáceres, Andréa Bezerra

**Affiliations:** ^1^Physical Activity Sciences Observatory (OCAF), Department of Physical Activity Sciences, Universidad de Playa Ancha, Valparaíso, Chile; ^2^Physical Education, Universidad Metropolitana de las Ciencias de la Educación, Ñuñoa, Chile; ^3^Department of Physical Education, Sports and Recreation, Universidad de La Frontera, Temuco, Chile; ^4^Physical Activity, Health and Education Research Group (AFSYE), Physical Education Pedagogy, Universidad Adventista de Chile, Chillán, Chile; ^5^School of Education, Universidad de Concepción, Los Ángeles, Chile; ^6^Physical Education School, Pontificia Universidad Católica de Valparaísio, Viña del Mar, Chile; ^7^Faculty of Education and Social Sciences, Universidad Andrés Bello, Viña del Mar, Chile; ^8^School of Education, Universidad Viña del Mar, Viña del Mar, Chile; ^9^Research Centre in Physical Activity, Health and Leisure, Faculty of Sports, Universidade do Porto, Porto, Portugal

**Keywords:** active break, school, children, physical activity, on-task behavior

## Abstract

**Background:**

Physical inactivity is prevalent in childhood. Schools can be an ideal context to promote the regular practice of physical activity since children spend there a large part of the day. In this sense, an emerging and current trend is active breaks at school. This article presents a study protocol that seeks to assess the feasibility and effectiveness of an intervention with active breaks (ACTIVA-MENTE program) in a school context on physical activity, on-task classroom behavior, and the physical activity enjoyment in schoolchildren.

**Methods:**

The protocol includes children aged 10 to 11 years. Two groups will be randomized (intervention and control groups). The intervention group will use the active break program, ACTIVA-MENTE, which consists of the application of a 4-min, 30-s video with moderate to vigorous-intensity physical activity. These breaks will be taken 6 times a day in the classes for 6 weeks. The total physical activity will be measured with accelerometers (Actigraph wGT3X-BT), the on-task behavior through the Direct Behavior Rating Scale and the level of enjoyment through the Physical Activity Enjoyment Scale.

**Discussion:**

Previous research reported that active breaks have positive results in physical activity levels. This study will be one of the few to design active breaks through videos without depending on the presence of a physical education teacher and it can also provide new findings on the effectiveness of an active break’s structure (e.g., types of exercises and intensity) on the indicated outcomes.

**Expected results:**

It is expected that the ACTIVA-MENTE program can be a suitable program for school settings, potentially increasing physical activity levels, and the commitment to the task, as well as be a pleasant moment for the students.

**Clinical trial registration:**

Clinicaltrials.gov, identifier NCT05403996.

## 1 Introduction

The benefits of physical activity for the quality of life of children and adolescents are well established, since it reduces the risk of chronic diseases and the symptoms of depression and anxiety, as well as improves the physical fitness, cognitive function and self-esteem (1–3). Additionally, physical activity is associated with better academic performance (4, 5) and behavior during school tasks (6, 7). In spite of this, worldwide reports reveals that more than 80% of the child population does not reach the recommended levels of physical activity (8). Moreover, the rates of physical activity implementation are expected to be reduced as the child ages from the first years of elementary school (9), worsening the physical inactivity status during growth and development phase. Of note, increasing children’s level of physical activity, especially moderate to high intensity, has significant implications for health and for accomplishing school tasks (10).

The integration of physical activity into the school routine is a key aspect to reduce sedentary behaviors. Elementary schools can be ideal settings for physical activity in children due to the amount of time spent at school and, in addition, due to the safe place with education professionals, who can direct not only the physical activity practice, but also include educational content (11). However, assigning more time for physical activity during the daily school often conflicts with the curricular demands, hiding physical activities promotion policies. Therefore, to make physical activity a priority in the school context, efficient strategies are needed (12). In this regard, active breaks are considered an emerging and suitable trend for physical activity integration into educational settings, fitting with the curricular timetable by interspersing extended periods of sitting with brief bouts of physical activity (13). It has been reported that active breaks are effective for increase the level of physical activity, for improve the classroom behavior (on-task behavior) (14) and for the enjoyment of movement (15).

Several factors are associated with active breaks practical application. The evidence shows that the teachers’ perception of the use of active breaks is usually positive (12, 16). Notwithstanding, it must be short, fast, suitable to be performed in the limited space available in the classroom, easy to implement (without sophisticated technological equipment) and must not imply a great time responsibility related to the teachers’ academic load (16). Without these characteristics, active breaks could have an adverse effect particularly on classroom behavior (17).

Recent reviews (11, 15, 18) proposed three approaches to implementing active breaks: (1) Active breaks as an interval/rest between two successive lessons; (2) Active breaks taken during the lesson; (3) Physical activity classes, with physical activity integrated into other subjects (e.g., mathematics, history, etc.). These multiple alternatives has shown to be limited due to a very broad school curriculum and the established priority for standardized tests (19). In spite of these limitations, interventions with active breaks implemented by the classroom teacher, or even using basic technology (e.g., audio) (20, 21) with duration of 10 to 20 min, two or three times a week twice a day (22, 23), or from three to five minutes every day (19, 24), show effectiveness to improve the physical activity level, academic performance, enjoyment, desire to learn, concentration and on-task behavior. Therefore, physical activity included in the daily instruction does not detract from academic performance, but it may actually enhance it (18). As a result, the academic classroom has the potential to be an integral component of a whole-of-school approach to physical activity that provides multiple health and learning benefits. Hence, is necessary to study the active breaks interventions because these can be an interesting potentially strategy to promote physical activity attractiveness for the students and teachers (25). In this sense, this work contributes to the existing gap regarding the incorporation and implementation of physical activity interventions in the classroom and in this way to increase the physical activity level, on-task behavior and enjoyment, since, unlike many school-based physical activity interventions, activity breaks require minimal disruptions in lessons (26). It is noteworthy that, there is not a consensus regarding the type of intervention (exercises type, amount, frequency and duration) and therefore more experimental evidence’s is required that addresses different intervention strategies (11, 27).

## 2 Aim

The aim of this study is to evaluate the effectiveness of an intervention with active breaks - ACTIVA-MENTE - on children’s physical activity levels, on-task classroom, and physical activity enjoyment.

## 3 Materials and methods

### 3.1 Study design

The study is carried under a quantitative method. This study uses an experimental design with a pre-and post-test. The sample will be divided into two groups: intervention and control. An active break program - ACTIVA-MENTE – will be applied to the intervention group in their respective classes during the school day for 6 weeks, while the control group will perform their regular school day (without any intervention).

**Figure 1:**
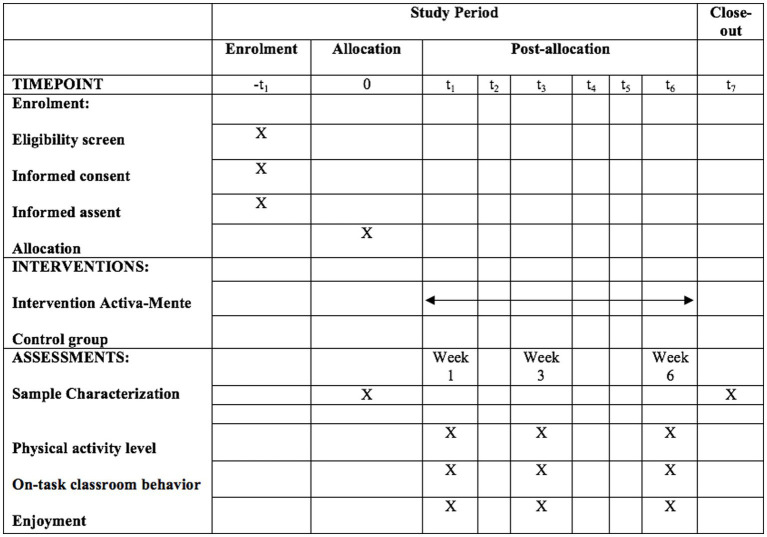
Standard Protocol Items: Recommendations for Interventional Trials (SPIRIT) (28). −*t*_1_, enrolment of the school and participants; 0, allocation of the groups and baseline assessments (high, weight, age); *t*_1_–*t*_6_, Intervention active breaks; t_1_, t_3_, t_6_ assessments; t_7_, post assessments (high, weight, age).

The protocol was developed in agreement with Standard Protocol Items: Recommendations for Interventional Trials (SPIRIT) and according to the verification guidelines of Consolidated Standards of Reporting Trials (CONSORT). Figure 1 provides a general description of the chronogram of enrolment, interventions and assessments.

### 3.2 Study sample

The study will be conducted at elementary schools in Valparaíso, Chile, from a low to middle low socioeconomic level (C_3_-D, respectively) (29). This group was selected in attempt to get a representative sample, since most of the Chilean population are classified with these socioeconomical level (29). The study sample consists in students of 10 to 11 years of age. The students who attend these schools spend approximately 8 hours a day at school (from 8:00 a.m. to 4:00 p.m.) with two recesses of approximately 15 min each (30 min altogether) and a longer rest for lunch of between 45 min and 1 h. For reasons of viability, the intention is to recruit two schools (one for intervention and another for control), from which a total sample size of 60 students is estimated.

#### 3.2.1 Inclusion criteria

Students aged 10 to 11 years old belonging to sixth grade elementary.

#### 3.2.2 Exclusion criteria

The excluded students will be those who present a class attendance below 90%, or students who present some health impediment when the active breaks are taken, or students with cognitive disability. These criteria are used since the effect of the intervention on the study variables (level of physical activity, behavior in the task and enjoyment) depends on the physical and psychological disposition for carrying out the active breaks of the students. Participants which match with exclusion criteria will be excluded only from the data analysis.

### 3.3 Sample size, recruitment, participants and allocation

Prior to starting the present study, a power analysis was performed (G*Power 3; Heinrich-Heine-Universität, Düsseldorf, Germany) to calculate the adequate sample size (F-test, effect size = 0.25, α error = 0.05, power = 0.95). According to this calculation, the participation of 54 students is estimated. Being an experimental study, the sample is not large, however it has great power and can be representative of this group of students (30).

The eligible school directors will be invited to participate initially by e-mail and then they will be contacted a week later by telephone. A researcher will meet with all the interested directors to explain the requirements for participation in the study. Then, be organized a presentation day to explain the project to the teachers and students. Those who agree in include their schools in the study will receive a statement in simple language and a consent form to be signed. The recruitment lasts 2 months and will be during the year 2024. Finally, the study setting is two primary school in the city of Valparaíso, Chile.

Once the consent of the school authorities has been obtained, the consent in writing for the elementary classroom teachers to participate will be obtained, since the intervention will be part of the daily class activities. Afterward, all the children in the participating courses will receive a document with the information regarding the program containing a declaration in simple language, a consent form for the parents or guardians, and an assent form confirm the children’s participation. After this authorization, the ACTIVA-MENTE program will be carried out with all the children in the classes, but only data from children which the parents’ consent and their assent of participation will be compiled. The participation in the program will be volunteer and if the children are free to decide to give in the program whenever they want.

**Figure 2:**
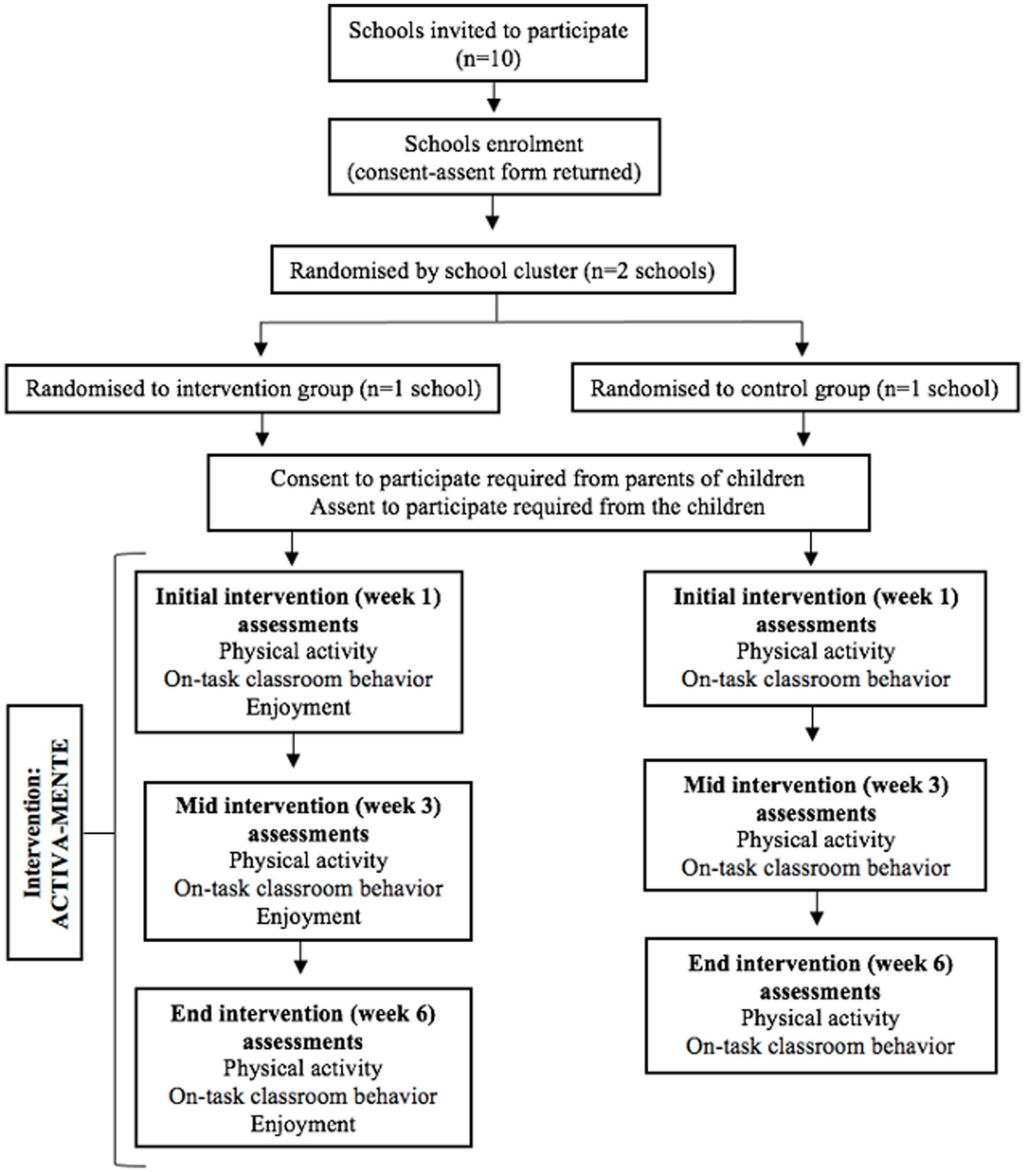
Flow chart of participants through the ACTIVA-MENTE study.

The randomization will be carried out by a blind researcher who has no contact with the schools or participants, using a random numerical sequence generated by the Oxford Minimization and Randomization (OxMaR) computer program (31). All the schools will be from areas of a middle socioeconomic position in a similar geographic location, avoiding great differences between the groups baseline characteristics (Figure 2). Possible differences identified between the intervention and the control groups will be adjusted in the statistical analyses to not influence the results.

### 3.4 Intervention

The ACTIVA-MENTE program is based on other programs previously reported in the literature which performed active breaks at school using audiovisual material (videos) (21, 24). A preliminary consultation was done with the teachers or with the head of student well-being in elementary schools that had previously applied the ACTIVA-MENTE non-systematically. One representative school was consulted, with responses from 11 other schools of similar demographic characteristics. The consultation was done to assess the viability of introducing regular active breaks in elementary classrooms, based on the perception of the students and principals regarding these interventions. Of these teachers, most (n = 10 of 12) positively valued the implementation of the program, considering it feasible to perform the designed active breaks in the program regularly (several times a day during the day), and indicated satisfaction by the teachers and students. In addition, most of them (n = 11–12) perceived improvements in students’ disposition and attention post-break session. Another important element of the ACTIVA-MENTE program is that the activities do not require a special technological element. The proposed activities only use equipment already available in the classrooms (e.g., audio, projector), attending teachers reported needs, such as fast and easy activities implementation.

### 3.5 ACTIVA-MENTE program

The program consists of teachers applying active breaks through previously recorded videos specially designed for the program. Online access to the program’s official website is free access: https://convivenciaparaciudadania.mineduc.cl/activamente/. These videos last 4 min and 30 s. This time is divided into 1 min of preparation (general explanation and indications), 3 min for 6 activities (e.g., jumps with feet together, skipping, jumping jacks, scissor kicks) of moderate-to high-intensity, i.e., over 60% maximum heart rate (HRmax) (32) the intensity will be monitored using the Polar OH1 device (Finland) and the rated perceived exertion scale (33) with a poster placed in front of the students when performing the break activities. Every activity is performed for 20 s with 10 s of recovery. During recovery time, the following activity is explained, the final 30 s are for the cool down (Table 1).

**Table 1:** Description ACTIVA-MENTE program.

Approaches	Organization: ACTIVA-MENTE video	Duration
In-person classes: students beside their desks	Beginning: general instructions	1 min
Teacher’s instructions to the class	Physical activity: 6 activities	3 min total
Instructions of the video guide	Execution of each activity (e.g., skipping, jumps)	20 s
Virtual classes: students are in front of a device (tablet, cell phone, computer)	Recovery and explanation of following activity	10 s
Instructions from the video guide	End: reincorporation to the other class activities	30 s
	Total time ACTIVA-MENTE	4 min 30 s

Min, minutes; s, seconds.

**Figure 3:**
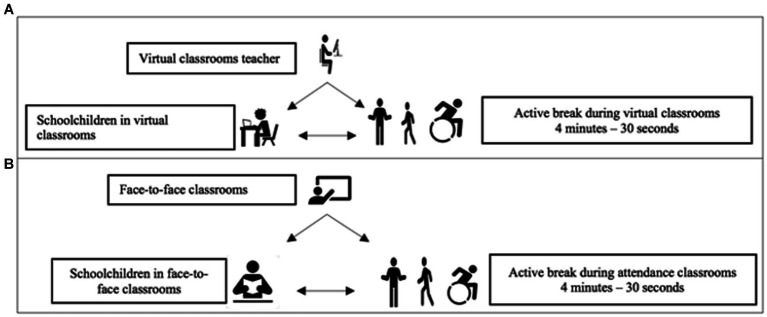
ACTIVA-MENTE model. **(A)** Active breaks during virtual classes for schoolchildren. **(B)** Active breaks during in-person classes for schoolchildren.

The online explanation of the activities execution is done by physical education teachers especially trained for this. The active breaks through videos are done in the middle of each class (for classes of 45 min the break is applied at minute 20), except physical education class, achieving six active breaks for day, during 6 weeks. There are 31 different videos that can be used interchangeably, the choice is up to the teacher (all the videos have the same structure). The activities suggested in the videos are based on previous studies (34–36) and the students can perform them standing up beside their desk. All the proposed activities can be adapted even for children with motor, auditory and/or visual disability (37). In addition, the program can also be implemented at online classes, using a platform that can share the video with the students (Figure 3).

### 3.6 Implementation, intervention fidelity and teacher training session

In order to improve the operation and adherence to the intervention protocol, a researcher will be present during the first 2 weeks to provide assistance. The research will provide the teachers with general support during the school visits to answer questions, adapt activities or give the teachers feedback. At the first week, both the researcher and the classroom teacher will implement the ACTIVA-MENTE activities together using the videos designed especially for the program. At week 2, the activities will be implemented only by the classroom teacher, while the researcher will observe and provide the feedbacks. Finally, at the third week, the classroom teacher will independently guide the ACTIVA-MENTE activities without the researcher presence. The teachers will be able to send e-mails or communicate at any time by telephone with the researchers if they have any concerns or they need more support. The use of the data collection instruments will be monitored by the researchers.

The fidelity of the program implementation will be evaluated by the use of accelerometers by children during school day in the first week (week 1), third week (week 3), and the last week of the intervention (week 6). The teachers will be instructed to complete a record of the daily application of ACTIVA-MENTE throughout the intervention period. Afterwards, a member of the research team will attend daily to collect the teachers’ records and to monitor the application of ACTIVA-MENTE. The data provided from the accelerometers will be compared with the teachers’ records at weeks 1, 3 and 6 of the intervention in order to verify the application of ACTIVA-MENTE. In the case of the teachers cannot perform the planned active break, they must indicate the reason. There will always be assistance staff available to remind the teacher of the application of active breaks.

One week before implementing the program, all the teachers will be trained for 45 min. The training session will be carried out in the schools by a researcher, who is a qualified elementary school teacher. The training session is designed to inspire and instruct the teachers with the necessary skills and knowledge to implement the ACTIVA-MENTE program. This training session content will include the importance to add physical activity times into the classroom routine; the evidence of current research that emphasizes the potential positive effect of active breaks to improve the physical activity levels and the on-task behavior; the explanation regarding the program; and finally, the instructions for program application. Since a researcher will be present during the first 2 weeks of implementation, extensive demonstrations of the active break activities will not be provided during the training session. After training session, the printed and the digital materials of ACTIVA-MENTE will be distributed, as well as a certification.

### 3.7 Procedures, outcomes measures and data collection

Three measurements will be assessed at weeks 1, 3 and 6: physical activity levels, on-task classroom behavior and enjoyment of physical activity. The implementation will be carried out by a group of research collaborators properly trained by the research team during four sessions previous to the assessment on the application of the instruments. The evaluators do not know the students and in the research, they only carry out the data collection.

#### 3.7.1 Primary outcome: physical activity level

ActiGraph wGT3X-BT accelerometers and the ActiLife 6 (United States) software will be used to measure the children’s level of physical activity. This device has proven validity and reliability to objectively measure the level of physical activity in children (38–40). The physical activity level will be only monitored during the school day.

#### 3.7.2 Secondary outcomes: on-task classroom behavior

Information will be compiled on the behavior of the students in the classroom individually using the Direct Behavior Rating Scale (41), and they will also be measured as a group by applying a modified version of the Classroom Behavior and Assets Survey-Teacher, which is designed to efficiently evaluate the teacher’s perception of the behavior of all the students in a class.

#### 3.7.3 Secondary outcome: physical activity enjoyment

The Physical Activity Enjoyment Scale (PACES) (42) will be individually applied to the participants. This is a scale that has reported good reliability in children (Cronbach’s alpha of 0.96) (43), and it has even been used in other studies with this population (44, 45). The questionnaire will be applied referring to “when I participate in the active breaks…”.

Physical activity: ActiGraph wGT3X-BT accelerometers and the ActiLife 6 software will be used to measure the children’s level of physical activity, since this device is proven to have the validity and reliability to measure the physical activity level objectively in this age group (38). The accelerometers will be used during the school day prior to the intervention, in weeks 1, 3 and 6, in both the intervention and control groups. The accelerometers will be handed out and collected from the child at school at the beginning and end of each school day.

The accelerometers will be placed at the beginning of every school day, since the device is programmed and activated by an internal clock from the software so that it begins to record for the established time. The accelerometers are placed using an elastic belt at the intersection of the right hip and waist (on the iliac crest), as this is the place next to the center of gravity (39). Data from the participants who do not fulfill what is requested for the recording (days and hours) will not be analyzed. A short duration epoch of 15 s will be used; because the pattern, more intermittent the children’s physical activity (46), frequency of 100 Hz. The classification of the daily level of physical activity will be: sedentary, light, moderate, vigorous and moderate-vigorous, established from counts per minute. The cut-off point used will be determined by Evenson’s equation for the ActiGraph wGT3X-BT, especially used for students’ task classroom behavior (47) (sedentary 0–100 counts^x^min^−1^, light 100–2,295 counts^x^min^−1^, moderate 2,296–4,012 counts^x^min^−1^, vigorous 4,013 counts^x^min^−1^).

On-task classroom behavior: Information will be compiled on the students’ behavior in the classroom individually and as a group. The observation will be done in weeks 1, 3 and 6, in the intervention and control groups. The members of the research team trained in the data collection instrument will make observations on the students’ behavior during a period of 15 min before and after participation in a break with ACTIVA-MENTE.

Individual classroom behavior will be measured using a tool adopted from the Direct Behavior Rating Scale (41). The Direct Behavior Rating Scale is a hybrid of behavior grading scales and direct observation. This observation tool requires the teacher to indicate for each child, on a scale from 1 (0%) to 10 (100%), the percentage of time they spend on the task, i.e., how committed they are to the task (e.g., listening to the teacher, writing, looking at instruction materials, etc.) during the observation period. This tool has evidence of reliability (r = 0.91) based on data from 617 elementary students from 44 classroom teachers (41).

Group behavior, otherwise, will be measured using a modified version of the Classroom Behavior and Assets Survey-Teacher, which is designed to provide the teacher’s perception of students’ behavior in class. The member of the research team trained in the instrument will indicate the proportion of the class that shows on-task behavior (as defined in the individual behavior assessment tool) during the observation period. The response options include: 0 (0 students), 1 (1–2 students), 2 (some students), 3 (approximately one quarter of the class), 4 (approximately half the class), 5 (approximately three quarters of the class), 6 (most of the class) and 7 (the entire class). Although there are few tools available to evaluate classroom behavior at class level, and a lack of information on reliability and validity, a modified version of this tool was used in a similar study, with evidence of reliability (alpha = 0.85) (13).

Physical activity enjoyment: The PACES will be applied in the weeks 1, 3 and 6, to the intervention group. PACES will be applied to the participants individually (42). This is a scale that has reported a high reliability in children (Cronbach’s alpha of 0.96) and it has even been used in other studies with children (45, 48). PACES consist of 16 items rated from 1 to 5, with 1 being “strongly disagree” and 5 being “strongly agree.” For example, positive items referred to statements such as “I enjoy it,” “I find it pleasurable,” and negative items had statements such as “I dislike it” or “It’s no fun at all.” The questionnaire will be applied referring to “when I participate in the active breaks…”.

As a general following up of the intervention, questionnaires will be used to collect information on the acceptability of the program by the directors, teachers and students in each school. They will be asked to report the facilitators and barriers to the implementation of ACTIVA-MENTE, as well as to suggest which component could be improved for implementation on a wider scale. Study participants will be asked about unexpected outcomes of the intervention. The field team will record positive and negative factors that affect the implementation to be able to optimize future interventions. This process will be done through semi-structured observations, where the research team will document the measured duration and fulfillment of the activities, the dynamic in the class or the activity, spaces, and others.

### 3.8 Ethics and dissemination: research ethics approval, consent and confidentiality

The study is designed according to the international ethical standards of the Declaration of Helsinki and was approved by the Scientific Ethics Committee of the Universidad de Playa Ancha (N° 005–2022). In addition, it follows the ethical standards for physical activity sciences and sports (49). A complete assessment about the protocol will be delivered to the parents or guardians and students during the study.

The parents or guardians will sign an informed consent allowing the students participation in the study. The students will also sign an assent form confirming they want to participate the study before the intervention be implemented.

Before, during and after the intervention and data collection, the potential participants’ personal information will be fully protected and will only be used for research purposes and dissemination of knowledge.

### 3.9 Data management, data access and dissemination policy

Data will be collected in the schools that are part of the study. This process will be done by a group of six research collaborators, who will provide the collected data to the principal investigator and to the co-investigator who will introduce them into two databases: Redcap and Dropbox. Then, the ordered data will be exported to a statistics program to execute the respective analyses.

The researchers responsible for this study will have access to the data. The anonymized data set will be at the disposal of the collaborating researchers and peer reviewers who require them through a virtual folder in the Dropbox application.

Once the study is finished, the results will be shared nationally via digital platforms (Webinar) of the Chilean Ministry of Education and internationally through conferences and seminars. For dissemination to the entire scientific community, articles will be published in scientific journals of the area.

### 3.10 Statistical analysis

All the statistical analyses will be performed using jamovi software (50). The data will be reported as the mean and standard deviation for both experimental and control groups. Multilevel mixed-effects linear regression models will be used to assess the impact of the group (intervention vs. control) on the average scores of the physical activity level, on-task behavior and enjoyment of physical activity during whole intervention. Each variable will be fit by baseline levels of the corresponding variable and grouping by classes. All the analyses will control for the levels of physical activity at the beginning. All the analyses will be stratified by sex. We will consider the results statistically significant only if the *p* value will be lower than 0.05. We will calculate the effect size to examine the magnitude of any mean differences.

## 4 Discussion

The main objective of this study is to examine the potential effectiveness of a physical activity program (ACTIVA-MENTE) implementation into the daily classroom, in the physical activity levels, on the children’s on-task behavior, and on the enjoyment of the program activities. Since the program will be implemented by instructed classroom teachers to simulate the application of the program in a real school context, the evaluation of the program viability and fidelity will be important characteristics of this pilot study.

A strength of this protocol is that it was developed with the contribution and orientation of current elementary teachers in order to fill a gap regarding the discrepancy between evidence and practice observed in previous studies (12, 51). For example, in the preliminary consultation, the teachers indicated that it is unlikely that active breaks of more than 5 min can fit in school routine. However, most of the active breaks studied throughout the time in a school context requires a duration of more than 10 min (11, 52). In fact, longer active breaks has also being described as not be suitable in a real school context, evidencing a greater limitation in these programs implementation (53, 54).

Recently, new studies which reduced the active break times, achieved positive increases in the level of physical activity and on-task behavior (6). It is expected that, following the current guidelines of active breaks and similarly shortening the periods of application, the ACTIVA-MENTE program might contribute with 27 min of daily physical activity of moderate to vigorous intensity. Another advantage is that ACTIVA-MENTE program facilitates the application for the teachers, given that the videos with the instructions for students all have the same structure, and therefore the teacher only needs to select the video with exercise demonstration, without increasing their workload. In this sense, the ACTIVA-MENTE program is pioneering in proposing an in-person and virtual model to apply active breaks.

Another strength of this study protocol is the measurement of the physical activity level using accelerometers, which give an objective measurement of the level of physical activity, as well as intensity. In addition, the data related to intensity will be complemented by the use of a heart rate monitor that will only be used during the application of ACTIVA-MENTE to provide a precise control of the intensity of the program’s activities. Furthermore, the teachers’ records regarding the physical activity performed are also included in the analysis. Together, both evaluations provide a differential and complete analysis in comparison with some studies which only assessed the physical activity level from the teachers’ reports. On the other hand, the application of this protocol seeks to investigate the integration of active breaks during the school day and their pedagogical impact, especially regarding students’ on-task behavior and enjoyment (55). In this line, some studies (11, 56) suggest that future investigations must focus on a rational and strategic application of interventions to evaluate their impact and effectiveness in the pedagogical process. In this sense, it is relevant that active break programs, such as ACTIVA-MENTE, include an assessment of student enjoyment related to the physical activities performed, which is another strength of the ACTIVA-MENTE study. Finally, ACTIVA-MENTE is designed to offer all children living in a situation of disability the opportunity to participate in the activities appropriate to their age and ability (3). This aspect is important, since the evidence shows that these disabled students not only enjoy physical activity, but also benefits themselves with this daily practice (57, 58). Specifically, this study can provide evidence regarding aspects, that are still not clear in active breaks, such as duration, intensity, types of exercise and inclusive methodology in a school context (59). The results of this study can provide useful information on the implementation and structure of future research that aims to address the feasibility and acceptance of intervention with active breaks, this is necessary, since active breaks are an emerging strategy to increase the physical activity level at school context and that has not yet been deepened (60).

## 5 Conclusion

The obtained results of applying the ACTIVA-MENTE intervention protocol are represented in increasing physical activity level, improving on-task commitment and being a pleasant moment for students. In this way, the results of this study will provide information about frequency, intensity, duration and types of exercises and of the effectiveness of the introduction of frequent, short active breaks into classroom routines, given the possible challenges in its application. A limitation of the study is that it is not designed for adolescents. It is advisable to generate new study protocols specifically aimed at secondary students due to their low levels of physical activity.

## Ethics statement

The studies involving humans were approved by Comite de Ética de la Universidad de Play Ancha. The studies were conducted in accordance with the local legislation and institutional requirements. Written informed consent for participation in this study was provided by the participants’ legal guardians/next of kin. Written informed consent was obtained from the minor(s)’ legal guardian/next of kin for the publication of any potentially identifiable images or data included in this article.

## Author contributions

TR-A, JI-M, and NA-F designed the study. TR-A, JI-M, JH-A, and JP-H extended the intervention. TR-A, JI-M, NA-F, NG-Á, AB, RZ-L, RY-S, GC, GR-C, and HC-B drafted and was involved in revising the manuscript critically for important intellectual content. All authors contributed to the article and approved the submitted version.
